# CSF-flow prior and after spinal tap test in patients with idiopathic normal pressure hydrocephalus—an exploratory study using real-time phase-contrast MRI

**DOI:** 10.3389/fnimg.2025.1665687

**Published:** 2025-12-12

**Authors:** Fiona Dierksen, Marielle Heide, Sabine Hofer, Felix Bernsdorff, Peter Dechent, Christian von der Brelie, Veit Rohde, Jan Liman, Mathias Bähr, Ilko L. Maier

**Affiliations:** 1 Department of Neurology, University Medical Center Göttingen, Göttingen, Germany; 2 Department of Cognitive Neurology, University Medical Center Göttingen, Göttingen, Germany; 3 Department of Neurosurgery, Johanniter-Kliniken Bonn, Bonn, Germany; 4 Department of Neurosurgery, University Medical Center Göttingen, Göttingen, Germany; 5 Department of Neurology, Paracelsus Medical School, Nürnberg, Germany

**Keywords:** CSF-flow, real-time phase-contrast MRI, normal pressure hydrocephalus, spinal tap test, iNPH

## Abstract

**Background:**

Neuroimaging plays a key role in the diagnostic workup of patients with idiopathic normal pressure hydrocephalus (iNPH). A flow void in the aqueduct - indicating increased cerebrospinal fluid (CSF) velocity - is a common, but unspecific finding. Aim of this study was to investigate CSF-flow characteristics in iNPH patients before and after spinal tap test (STT) using novel, real-time phase-contrast magnetic resonance imaging (RT-PC MRI).

**Methods:**

We included consecutive patients with clinical signs of iNPH being electively admitted for diagnostic workup, including neurological examination, conventional MRI and STT. RT-PC MRI and clinical examination were performed before and within 24 h after STT. CSF-flow volumes were determined at five regions in the inner and outer CSF spaces.

**Results:**

Fifteen patients with suspected iNPH and five age-matched healthy controls (HC) were included. Baseline RT-PC MRI revealed elevated CSF-flow volumes in the inner ventricular system of iNPH patients compared to healthy controls, being detectable predominantly in the third ventricle (iNPH vs. HC: 15.93 ± 7.01 mL vs. 6.58 ± 2.99 mL, *p* = 0.020). There was a positive correlation between the Evans Index and CSF-flow in the third ventricle (r = 0.586, *p* = 0.017), cerebral aqueduct (r = 0.639, *p* = 0.006) and the fourth ventricle (r = 0.649, *p* = 0.007). There was no statistically significant change of CSF-flow volumes before and after STT in the iNPH-group.

**Conclusion:**

RT-PC MRI provides a promising, non-invasive approach for evaluating CSF-flow in iNPH. Baseline CSF-flow volumes were elevated in the inner ventricular system, particularly in the third ventricle, and correlated with ventricular enlargement, suggesting that increased CSF-flow may reflect disease progression rather than therapeutic response. However, in contrast to clinical tests, the lack of change of CSF-flow after STT limits its utility for patient selection for ventriculo-peritoneal-shunt implantation.

## Introduction

The idiopathic normal pressure hydrocephalus (iNPH) is characterized by a triad of clinical symptoms: a slowly progressive gait disturbance, urinary incontinence and cognitive impairment that develop over time (Relkin et al., 2005). Most patients initially present with gait disturbances as the first symptom. The diagnosis of the iNPH is based on three criteria: (1) typical clinical features, (2) specific neuroimaging findings and (3) clinical improvement following a spinal tap test (STT) (Krauss and Halve, 2004). A significant improvement in gait after the removal of large volumes of cerebrospinal fluid (CSF), coupled with the exclusion of secondary causes, may indicate the need for a ventriculoperitoneal shunt (VP-shunt) implantation (Tisell et al., 2011).

However, the risk of misdiagnosis in clinical practice is high due to overlapping clinical and MRI features with other conditions such as Alzheimer’s and Parkinson’s disease, subcortical arteriosclerotic encephalopathy and multi-infarct syndrome (Tullberg et al., 2002; Bech-Azeddine et al., 2007; Krauss et al., 1997; Hebb and Cusimano, 2001). This diagnostic overlap is clinically important because accurate identification of iNPH is essential to select patients who may benefit from VP-shunting, avoiding unnecessary procedures in patients with other neurodegenerative disorders. Recent advances in MRI-based CSF-flow measurements, including aqueductal stroke volume and real-time phase-contrast imaging, have been proposed as quantitative biomarkers to improve diagnostic accuracy and predict shunt responsiveness (Navarro-Ballester, 2025; Rivera-Rivera et al., 2024; Shojaeianforoud and Lahooti, 2025). As a result, the long-term benefit of VP-shunt implementation varies widely, ranging from 29 to 80% (Halperin et al., 2015). The variability may be attributed to placebo effects, secondary causes of NPH, patient-valve-mismatch or due to the chronic progressive processes of the disease. Despite these limitations, a positive response to STT remains a widely accepted diagnostic tool, although its pathophysiological mechanism is not fully understood (Halperin et al., 2015). The greatest improvements in gait are typically seen within 24 to 48 h after STT and are predictive of a positive response to VP-shunt implantation (Schniepp et al., 2017).

Neuroimaging plays a key role in supporting the diagnosis of iNPH. On MRI as well as CT scans the iNPH is characterized by an enlargement of the lateral and third ventricles, a widening of the Sylvian fissure and crowding at the vertex. Other morphological hallmarks include an Evans index greater than 0.33, and a narrowed callosal angle (typically between 40 and 90°), reflecting disproportionate dilation of the ventricular system relative to subarachnoid spaces (Damasceno, 2015). Additionally, periventricular white matter changes may occur due to transependymal CSF leakage, likely being caused by intermittent peaks of the intracranial pressure (ICP) (Kockum et al., 2018). Beyond structural markers, CSF-flow – particularly in the aqueductal region – have emerged as an important diagnostic and prognostic tool. Although early studies focused on qualitative signs such as aqueductal flow voids on T2- and proton density-weighted sequences, these are now considered unreliable due to their frequent appearance in healthy individuals on high-field MRI and their dependence on imaging parameters. Current methods use phase-contrast MRI to measure aqueductal stroke volume, the average CSF-flow through the cerebral aqueduct during the cardiac cycle, which reverses direction between systole (caudal flow) and diastole (rostral flow). Respiration also influences CSF dynamics by modulating intracranial and intraspinal pressure, further contributing to flow direction and velocity. In iNPH, this has been shown to result in an increased flow void on proton density-weighted MRI, that often extends beyond the aqueduct into the third and fourth ventricles (Damasceno, 2015).

Elevated stroke volumes of CSF and peak flow rates have been associated with better outcomes following VP-shunting (Krauss et al., 1997). A flow rate >24.5 mL/min has shown a specificity of 95% for iNPH (Al-Zain et al., 2007; Manley et al., 2009), while a CSF stroke volume ≥42 μL has been suggested to predict favorable shunt response (Bradley et al., 1996). However, these thresholds vary between studies, and inter-institutional differences in scanner calibration necessitate center-specific reference values (Bradley, 2008; Ringstad et al., 2015). And some studies even doubt the usefulness of aqueduct stroke volume in patient selection for VP-shunting at all (Ringstad et al., 2015). Notably, reduced aqueductal stroke volume in advanced disease stages – despite ongoing clinical symptoms – has been linked to cerebral atrophy and may predict limited benefit from surgical intervention (Scollato et al., 2008).

Real-time phase-contrast MRI (RT-PC MRI) is an advanced imaging technique that enables rapid, high-resolution assessment of CSF-flow dynamics, both intracranially and extracranially (Sahoo et al., 2024). It allows the evaluation of flow velocities and flow volumes independent of the cardiac cycle during free breathing and has been investigated in healthy subjects with CSF-flow patterns (Chen et al., 2015). The present study aims (1) to investigate possible differences in CSF-flow volumes in the inner- and outer CSF spaces between patients with iNPH and (2) to investigate the change in CSF-flow volume after STT and its association to clinical improvement and VP-shunt indication. We hypothesize that a reduction of CSF volume after STT might result in reduced CSF-flow volumes and therefore serve as a predictor of clinical improvement and potential responsiveness to VP-shunting.

## Methods

We conducted a single-center, prospective study involving patients with suspected iNPH, who were evaluated before and after undergoing a STT. Five age-matched healthy individuals served as a control group (compared within Table 1). Patients were recruited from the Departments of Neurology and Neurosurgery at the University Medical Center Göttingen (UMG), Germany. This was an exploratory, hypothesis-generating study and was not powered for confirmatory hypothesis testing. All analyses are therefore primarily descriptive; inferential statistics are reported as exploratory. RT-PC MRI was conducted at the Biomedical NMR Research Facility at the Max Planck Institute of Multidisciplinary Sciences and the Department of Cognitive Neurology at UMG using the same protocol.

**Table 1:** Baseline characteristics of iNPH-group and age-matched healthy control group.

Baseline characteristics	iNPH (*n* = 15)	HC (*n* = 5)	*p*-value
Age (years, mean ± SD)	77.8 ± 6.5	70.4 ± 9.6	0.066
Sex male [*n* (%)]	12 (80)	1 (20)	
Cognitive impairment [*n* (%)]	13 (86.6)	1 (20)	
Urge or urinary incontinence [*n* (%)]	8 (53.3)	0 (0)	
Gait disturbance [*n* (%)]	15 (100)	0 (0)	
Hakims triad fulfilled [*n* (%)]	7 (46.6)	0 (0)	
CSF pressure (mean cmH_2_O ± SD)	18.4 ± 4.6	n.a.	
CSF removal (mean ml ± SD)	31.3 ± 5.3	n.a.	
Time between spinal tap test and second MRI [Mdn hours (IQR)]	22.5 (17–24)	n.a.	
Evans index (mean ± SD)	0.37 ± 0.03	0.28 ± 0.04	**0.002**
Callosal angle (mean ± SD)	86.9 ± 24	142.3 ± 16.6	**0.003**
Fazekas-scale [Mdn (IQR)]	1 (0–2.5)	1 (0–1)	0.497
Shunt-indication after spinal tap test [*n* (%)]	7 (46.6)	n.a	
Clinical tests at the baseline			
TUG test [Mdn s (IQR)]	16.5 (11.8–36)	7.3 (6.4–9.4)	**0.002**
30-MWT [Mdn steps (IQR)]	60 (48–68.5)	41 (37–46.5)	**0.028**
30-MWT [Mdn s (IQR)]	37.6 (28.1–57.5)	20.5 (17.8–23.7)	**0.002**
MoCA-Score [Mdn P (IQR)]	22 (19.5–24)	28 (22–28.5)	0.065
TMT A [Mdn s (IQR)]	63.8 (46.5–91.3)	38.1 (24–56.6)	**0.046**
TMT B [Mdn s (IQR)]	193.5 (123.9–282.3)	94.2 (51–181.8)	0.078

This table presents the clinical and radiological characteristics of patients with idiopathic normal pressure hydrocephalus (iNPH) and the healthy control group. Significant differences were observed in pathological parameters of iNPH including the Evans index, corpus callosal angle and performance in gait and cognitive assessments. CSF: cerebrospinalfluid; HC: Healthy Controls; TUG-Test: Timed-Up-and-Go-Test; 30-MWT: 30-meter-walking-test; MoCA: Montreal-Cognitive-Assessment; TMT: Trail-Making test; P: Points; Mdn: Median; IQR: interquartile range; n.a.: not applicable. Values in bold indicate statistical significance at *p* < 0.05.

Inclusion criteria were based on the national diagnostic guidelines for iNPH by the German Society for Neurology. The presence of a gait disturbance, as a mandatory feature of Hakim’s triad, was required, along with at least one additional symptom (cognitive impairment or urinary incontinence). Furthermore, conventional cranial MRI of iNPH subjects was performed prior to study inclusion and showed disproportionate ventricular enlargement. MRI scans were only performed in patients who were able to successfully complete the respiratory protocol performing three pre-scan training runs. Compliance during scanning was monitored visually by the investigator by judging the changes of the SpO₂ curves. Evans-Index > 0.3 was viewed pathological as quantitative criteria of iNPH (Damasceno, 2015). Exclusion criteria included contraindications for MRI, inability to complete the respiratory gating protocol, or evidence of secondary causes of hydrocephalus.

All included patients underwent two RT-PC MRI scans: one prior to and one following the STT. The second MRI was conducted within 65 h after STT (median: 22.5 h; IQR: 17–24 h). For the STT, the lumbar puncture was performed with the patient in an upright position. After needle insertion, the patient was repositioned into the lateral decubitus position for intracranial pressure measurement, after which the CSF was removed. After measurement of CSF opening pressure an average CSF-volume of 31.3 mL ± 5.3 mL was drained. All CSF samples were analyzed for cell count, lactate, total TAU/phosphor-TAU and the ß-amyloid 40/42 ratio – all of these values were within the normal limits.

The study was approved by the Ethics Committee of the UMG (approval number: 29/6/18) and written informed consent was obtained from all participants.

### Real-time phase-contrast MRI

**Figure 1:**
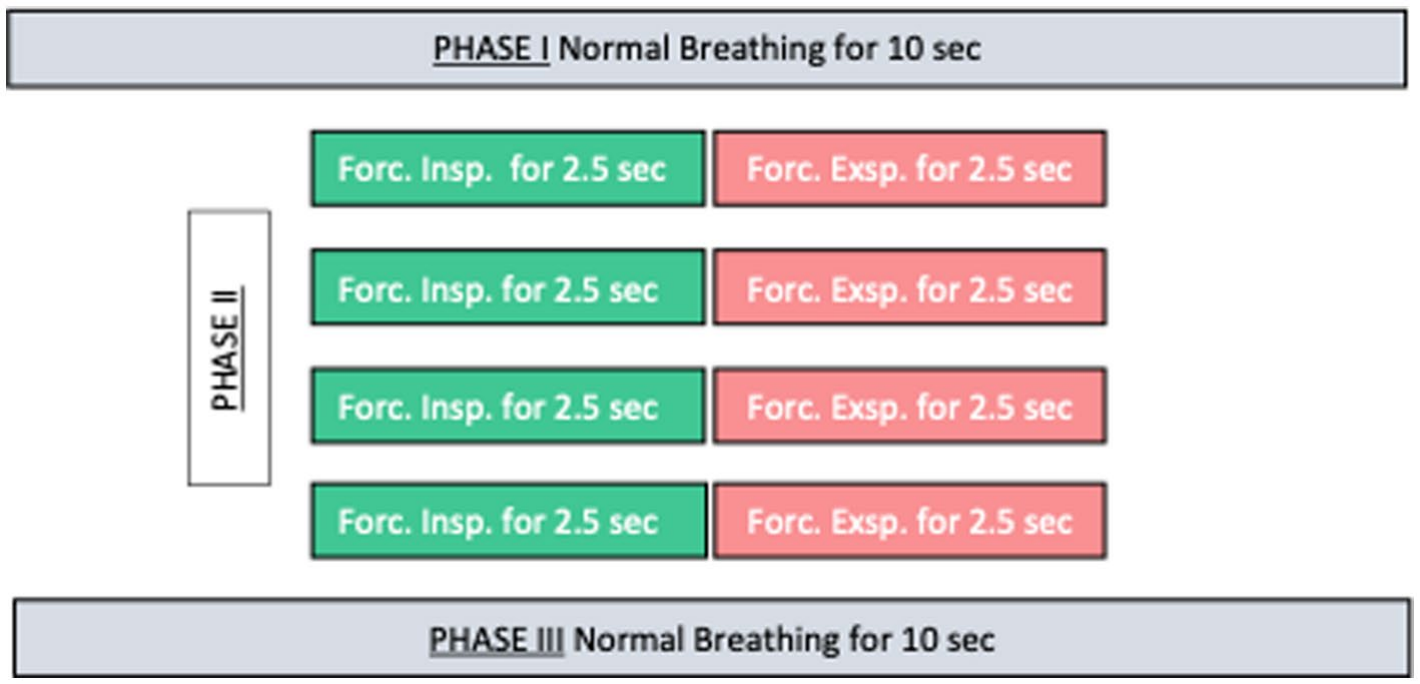
Respiratory protocol consisting of 3 phases. During Phase I and Phase III normal breathing and Phase II four cycles of forced inspiration and expiration for 2,5 s each.

The measurement was performed using a 3 Tesla MRI system (Magnetom Prisma, Siemens Healthineers, Erlangen, Germany) equipped with a 64-channel head coil. To optimize visualization and quantification of CSF-flow, patients followed a breathing protocol during image acquisition including both phases of normal breathing (Phase I and III) as well as a phase of 4 times forced in- and expirations for 2.5 s, respectively (40 s in total, see Figure 1). This protocol had been practiced before the scan with the patients to ensure comparable performance. Standard pulse oximetry has been used to visually control for compliance of the protocol during the scan. If patients did not adhere properly to the protocol, the scan was repeated.

**Figure 2:**
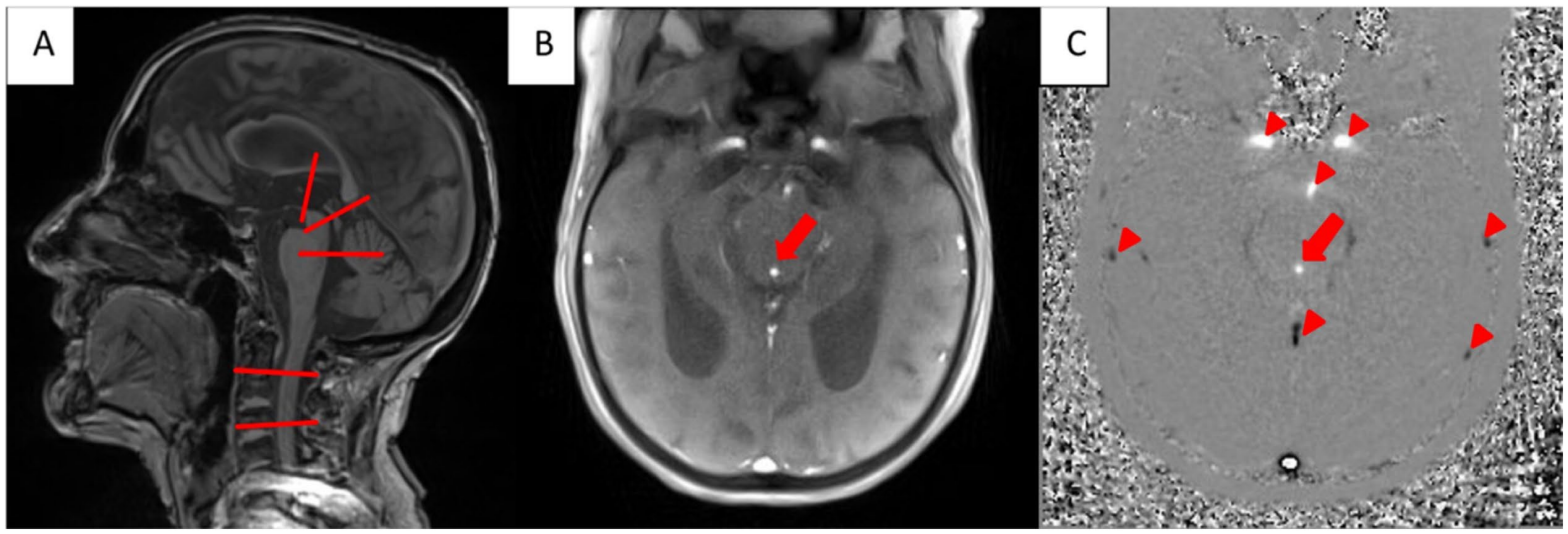
**(A)** T1-weighted MRI in sagittal plane. The red lines mark the ROIs for the CSF-flow measurements: third ventricle, aquaeductus mesencephalic, fourth ventricle, extracranial: subarachnoid space (second and fourth cervical vertebra). **(B)** Anatomical magnitude real time phase contrast MRI in a transversal plane showing CSF-flow in the aquaeductus mesencephalic (red arrow). **(C)** Corresponding phase contrast image with cranially directed CSF-flow (blue arrow). Note that in the phase contrast image cranially directed flow is visualized hyperintense and caudally directed flow hypointense (Arrowheads mark the carotid arteries and the basilar artery (hyperintense) and draining veins/sinus).

The CSF-flow measurements were conducted at five slice positions for through plane flow, which were identified within the median sagittal-, axial- and coronary plane of a T1-weighted image. These slice positions were located at the aqueduct, 3^rd^ and 4^th^ ventricle as well as at the level of C2 and C4 perpendicular to the supposed CSF-flow direction (See Figure 2A). To ensure consistent localization across scans, the slice positions for through-plane flow measurements were saved and matched to the second scan following the STT.

CSF-flow was measured using magnitude images of the anatomical structures for reference and phase-contrast images that provided velocity and flow direction information. Representative magnitude and phase-contrast image series used for CSF-flow measurements are shown in Figures 2B,C as well as are available in the Supplementary Videos 1, 2.

The RT-PC MRI is based on a high under sampled radial fast low angle shot (FLASH) sequence in combination with a model-based reconstruction technique offering high spatiotemporal resolution. The following measurement parameters were used: repetition time (TR) of 5.68 ms, echo time (TE) of 4.61 ms, a flip angle 10°, echo asymmetry 30% (50% symmetry), slice thickness of 5 mm, radial k-space scanning with 11 radial spokes per image, a field of view (FOV) of 192 × 192 mm^2^ with a base resolution of 256 resulting in an in-plane resolution of 0.8 × 0.8 mm^2^. The velocity encoding strength (VENC) was set to 10 cm°s^−1^ according to the expected velocity range. The CSF-flow (ml/s) was calculated by multiplying the size of the measurement area (mm^2^) with the velocity. Regions of interest have been defined using CaFur (CaFur, Fraunhofer MEVIS, Bremen, Germany), a tool specifically dedicated for processing dynamic MR imaging, in the above-mentioned slice positions including the areas of CSF-flow being identified on the magnitude images. CaFur enables automated segmentation of CSF spaces and extraction of flow parameters across the entire time series. Enhanced spatial and temporal resolution of the magnitude images and velocity maps, combined with dynamic modeling of deformation fields, allows for accurate delineation of CSF-flow regions and minimizes partial volume effects. Derived flow metrics include peak and mean velocities, cross-sectional areas, and flow volumes at predefined ROIs (Chitiboi et al., 2017). For this study, the total amount of cranial and caudal flow (ml) during the breathing protocol (40 s) within the predefined ROI has been determined and analyzed. To ensure protocol consistency, patients completed supervised respiratory training prior to scanning, practicing the sequence of normal and forced breathing. During image acquisition, adherence to the protocol was visually monitored by the MRI technician and supported by continuous SpO₂ monitoring; however, no quantitative respiratory belt or spirometer recording was performed. Motion correction was achieved through visual inspection, and scans showing visible motion artifacts were excluded from analysis. Regions of interest were placed by the same examiner following a standardized procedure; in cases of uncertainty, a second investigator was consulted to reach consensus.

### Examination of gait and cognition

All patients in the iNPH group and the healthy controls underwent multiple test batteries regarding gait and cognition. To assess gait impairment, the Timed Up and Go (TUG) test was performed, where patients were instructed to stand up from a chair, walk three meters, turn around, and sit back down. The time was recorded and the limitations were categorized as follow: a time <10 s was considered normal, 10–19 s as moderate, 20–29 s as functionally relevant and >30 s as severe impairment (Podsiadlo and Richardson, 1991). A second test involved a 30-meter walk, with measurements of both time and step count (Singh and Crockard, 1999). These tests were conducted prior to STT and again within 24 h after STT. Previous studies have demonstrated a correlation between these tests and the diagnosis of iNPH (Ishikawa et al., 2016). To assess the cognitive function, the Montreal Cognitive Assessment (MoCA) (Nasreddine et al., 2005) and both parts of the Trail Making Test (A and B) (Tombaugh, 2004) were administered. Improvement of motor skill changes before and after the tests were calculated using the following formula (Schniepp et al., 2017):


$$\frac{\text{results prior to}\;\mathrm{STT}-\text{results after}\;\mathrm{STT}}{\text{results before}\;\mathrm{STT}}\times 100$$

An improvement of 20% in TUG and/or 30MWT was considered clinically significant and defined as responder, while patients with <20% improvement were classified as non-responders (Schniepp et al., 2017). This threshold serves as a clinical indicator for shunt candidacy. In cases of a deterioration in any parameters, a zero-percentage change was recorded. Urinary incontinence was assessed based on the medical history.

### Statistical evaluation

Statistical analysis was conducted using GraphPad Prism 6.0 (GraphPad Software, San Diego, California, USA). Baseline characteristics are presented as the mean ± standard deviation (SD) for normally distributed and as the median (Mdn) ± interquartile range (IQR) for non-normally distributed data. The *t*-test was applied to normally distributed data. Following recommendations for exploratory studies, a Bonferroni adjustment for *p*-values was not performed. Given the number of comparisons performed across multiple ROIs and correlations, the risk of type I error inflation must be considered; thus, all results should be interpreted as exploratory and descriptive rather than confirmatory. The correlation between radiological and clinical scores was assessed using bivariate Pearson correlation if normally distributed and Spearman’s rank correlation, if not. *p*-values <0.05 were considered statistically significant.

## Results

Given the exploratory and hypothesis-generating nature of this study, the following results should be interpreted as descriptive and preliminary, intended to identify potential trends rather than establish definitive statistical associations.

### Characteristics of patients

The mean age of the patients with iNPH was 77.8 ± 6.5 years, compared to 70.4 ± 9.6 years in the healthy controls. All individuals in the iNPH group exhibited a disturbance of gait. Thirteen (86.7%) patients additionally demonstrated cognitive impairment (defined as a MoCA score below 26 points) and 8 (53.3) had an urge- or urinary incontinence. Cognitive impairment was present in one individual (20%), no gait disturbances or urinary incontinence were observed in the HC group (Table 1).

The Evans-Index tended to be higher and the callosal angle tended to be lower in the iNPH group (*p* < 0.05). Motor- and cognitive performance were impaired in the iNPH group as compared to the HC group with significantly poorer performance in the TUG- and 30-meter-walking-test as well notably lower performance in the MoCA and Trail-Making test.

### Comparison of CSF-flow volume in the iNPH group prior to STT vs. HC group

**Figure 3:**
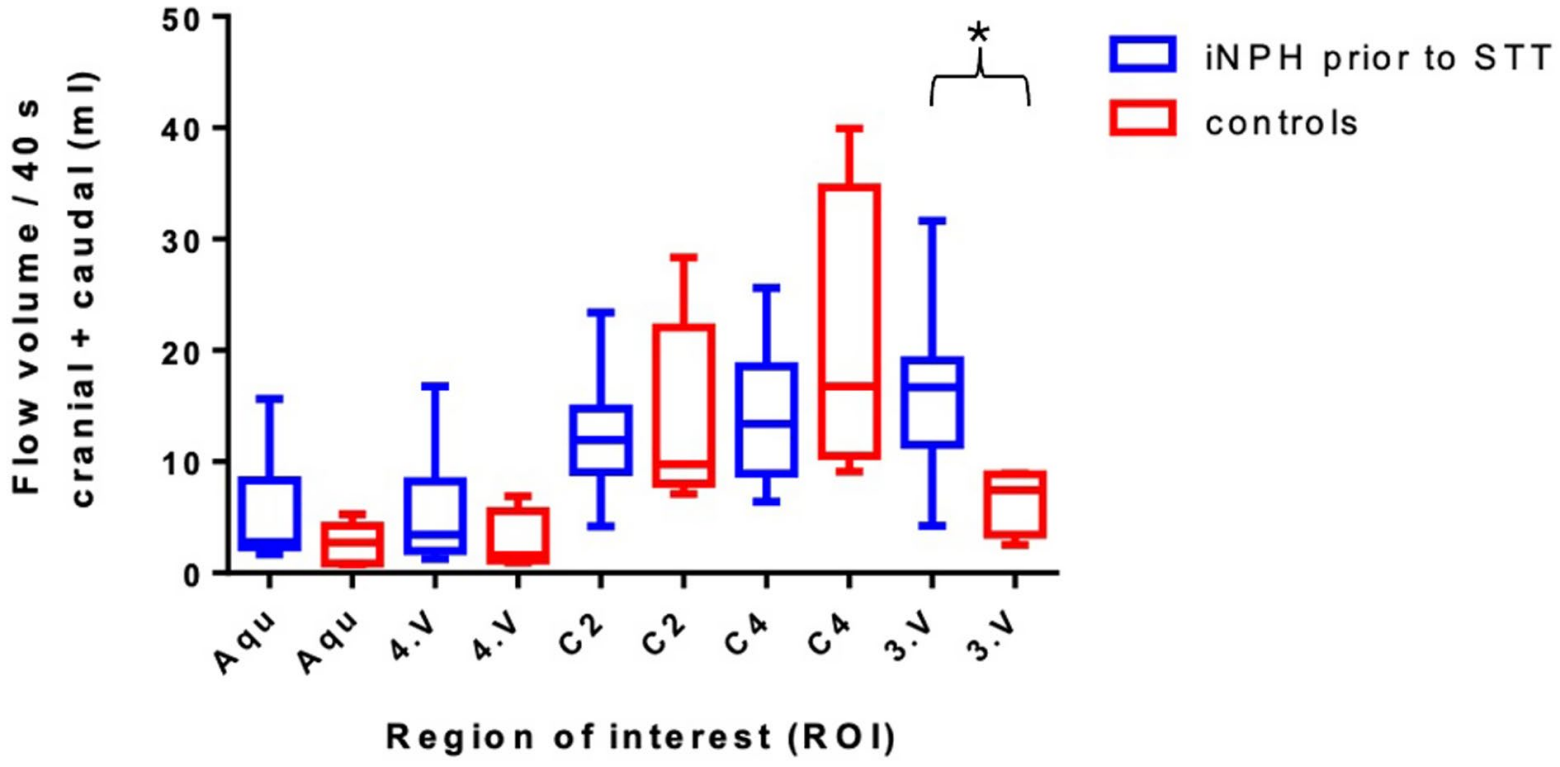
Comparison of the total flow volume in ml of iNPH vs. HC at the regions of interest with their standard deviations. Aqu – cerebral aqueduct, 4. V – 4. ventricle. C2 – second cervical vertebra. C4 – fourth cervical vertebra, 3. V – third ventricle. *p < 0.05; **p < 0.01; and ***p < 0,001.

First, the CSF-flow volumes were compared between patients with iNPH prior to the STT and HC. Across all subjects, CSF-flow volumes showed considerable inter-individual variability, with particularly pronounced standard deviations observed in the iNPH group. Figure 3 presents box plots illustrating flow volumes across all regions of interest for both groups. Regarding the inner CSF spaces, patients with iNPH demonstrated higher flow volumes compared to HC, notably in the aqueduct (range across the entire cohort: 1.69–15.65 mL; iNPH vs. HC: 5.19 ± 4.35 mL vs. 2.58 ± 1.85 mL, *p* = 0.214), the third ventricle (range 4.22–31.66 mL; iNPH vs. HC: 15.93 ± 7.01 mL vs. 6.58 ± 2.99 mL, *p* = 0.020) and the fourth ventricle (range: 1.29–16.75 mL; iNPH vs. HC: 5.57 ± 4.99 mL vs. 2.75 ± 2.77 mL, *p* = 0.299). To further evaluate the diagnostic performance of third ventricle CSF-flow volumes in differentiating iNPH patients from healthy controls, we performed a ROC analysis. The analysis yielded an AUC of 0.883 (95% CI: 0.724–1.0), indicating good discriminatory ability. The optimal cut-off value was 10.23 mL, corresponding to a sensitivity of 0.80 and specificity of 1.00 (Supplementary Figure 3).

Flow volumes in the outer CSF spaces showed even greater variability but no clear differences between groups. At the level of the second cervical vertebra, flow volumes ranged from 4.2–23.39 mL (iNPH vs. HC: 12.68 ± 5.87 mL vs. 13.99 ± 8.67 mL, *p* = 0.705). At the level of the fourth cervical vertebra, volumes ranged from 6.39–25.61 mL (iNPH vs. HC: 13.92 ± 5.87 mL vs. 20.65 ± 13.46 mL, *p* = 0.152).

### Comparison of change of CSF-flow volumes prior- and after STT in iNPH patients

**Figure 4:**
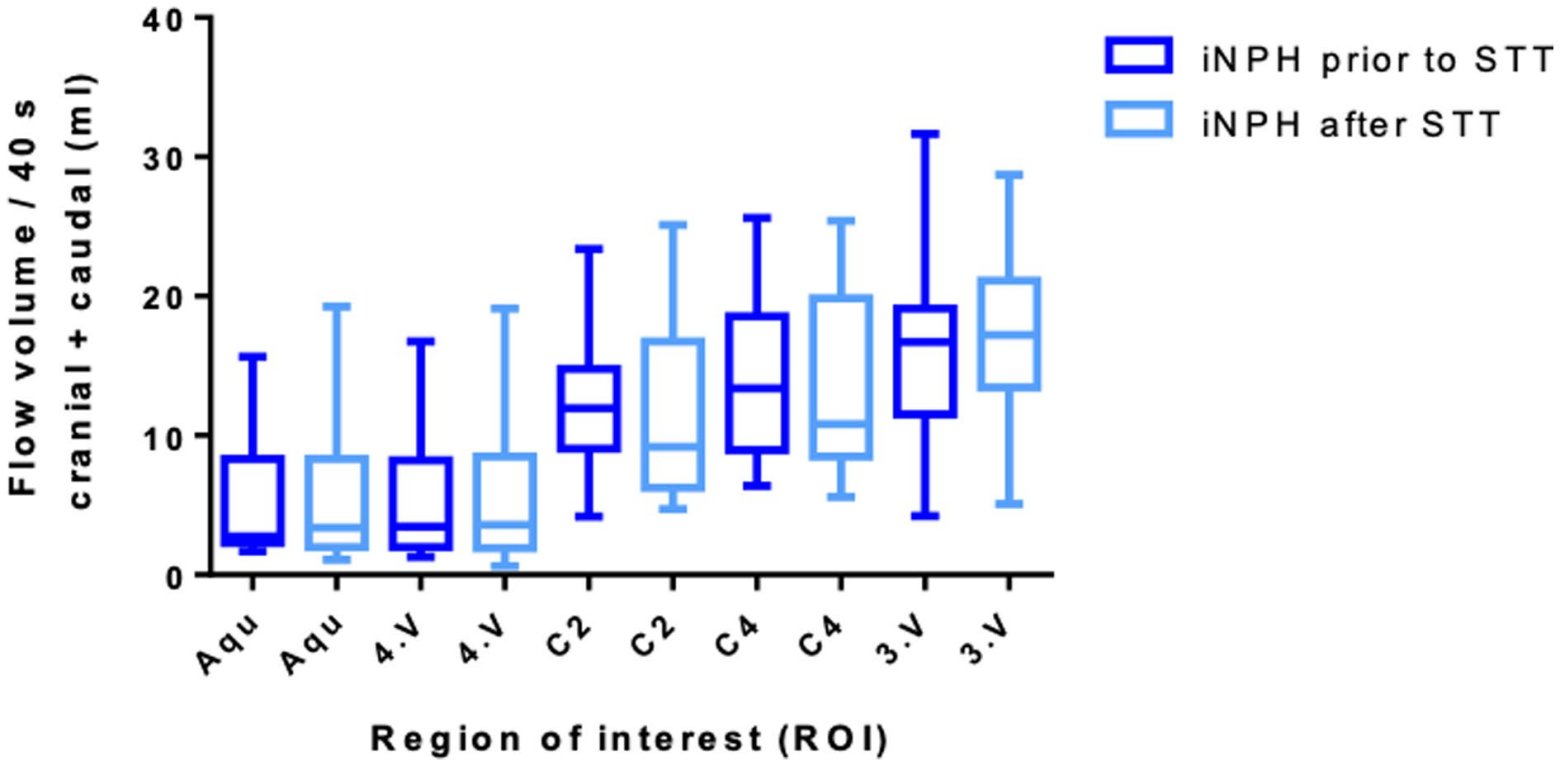
Comparison of the total flow volume in ml of iNPH group prior and after STT with their standard deviations. Aqu – cerebral aqueduct, 4. V – 4. ventricle. C2 – second cervical vertebra. C4 – fourth cervical vertebra, 3. V – third ventricle.

In the next step, CSF-flow volumes were compared prior to- and after the STT in patients with iNPH. As illustrated in Figure 4, no clear differences were observed in total (cranial + caudal) flow volumes across all measured regions. Specifically, the mean flow volume before and after the STT was compared using paired *t*-tests. The *Δ* flow volume refers to the difference between the post-STT to pre-STT mean flow volumes within the iNPH group. The mean change (Δ flow volume, in ml) were as follows: 0.61 mL in the aqueduct (*p* = 0.740), 0.28 mL in the fourth ventricle (*p* = 0.886), −0.72 mL at the C2 level (*p* = 0.751), −0.25 mL at the C4 level (*p* = 0.917), and 1.15 mL in the third ventricle (*p* = 0.673). The comparison prior and after STT in the aqueduct is illustrated in Supplementary Video 1 and in the third ventricle in Supplementary Video 2.

Notably, although these changes did not reach statistical significance, a trend toward increased CSF-flow was observed in the intracranial regions (aqueduct, third and fourth ventricles) following STT, while the extracranial regions (C2 and C4 levels) appeared to show slight decreases in mean flow volume. This may suggest a potential shift in CSF dynamics post-STT, with increased flow in the ventricular system following the STT possibly indicating transient alterations in intracranial compliance.

Several physiological mechanisms may explain why CSF-flow measured with RT-PC MRI did not change appreciably after STT in our cohort. First, intracranial compliance in iNPH is often reduced, meaning pressure changes may not translate into sustained bulk flow alterations (Mase et al., 2008). Second, venous outflow pathways play a critical role in modulating CSF dynamics; constricted venous drainage has been associated with increased CSF pulsatility at the aqueduct and reduced absorption capacity, potentially buffering or offsetting expected flow changes following a STT (Shojaeianforoud and Lahooti, 2025; Beggs et al., 2019). Third, recent work differentiating cardiac- vs. respiratory-driven CSF motion shows that these drivers may be differentially affected, and respiration components may show more variability or be less responsive to acute changes induced by STT (Karki et al., 2025).

### Comparison of CSF-flow volumes in patients with- and without clinical response to STT

**Figure 5:**
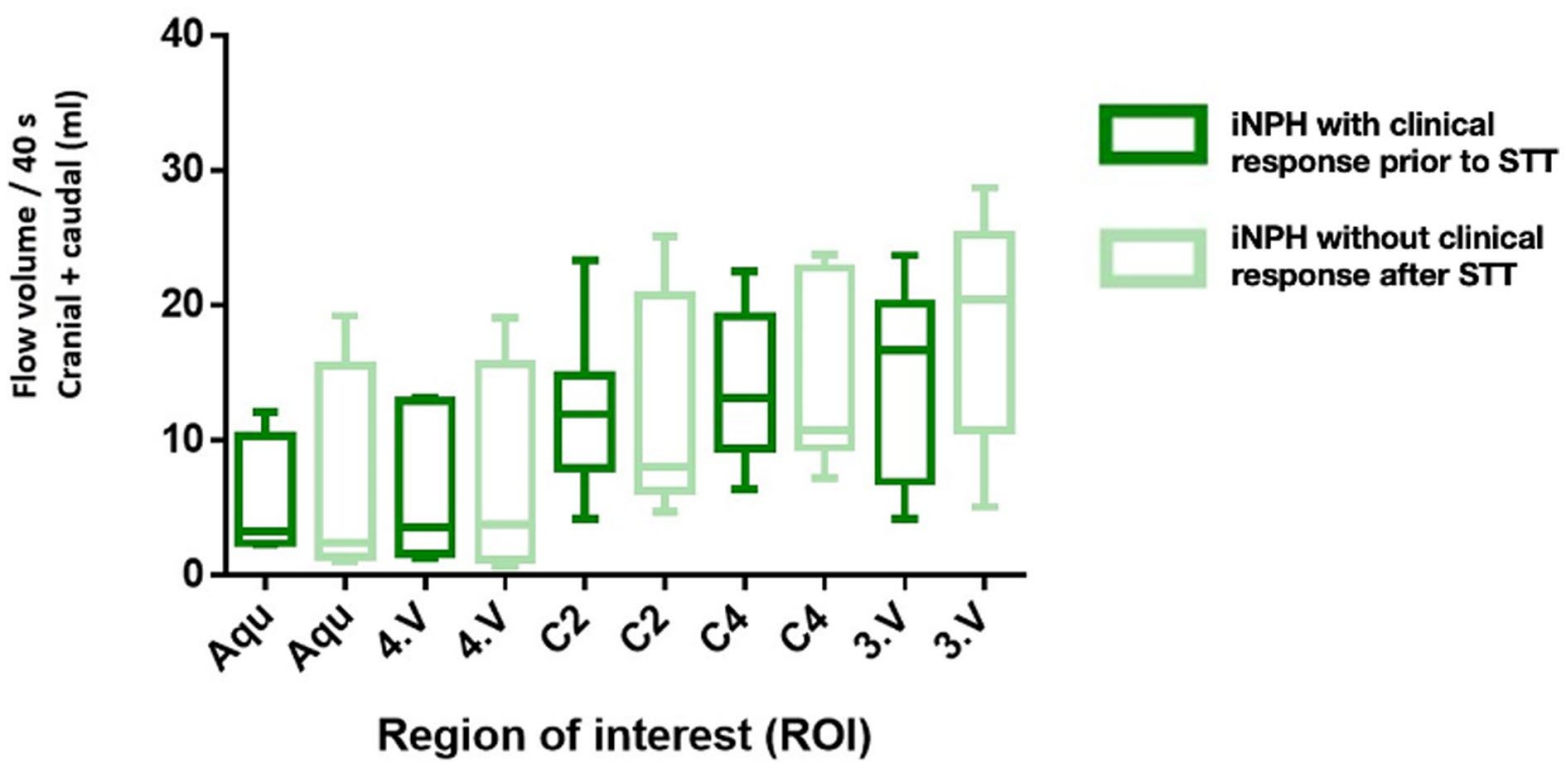
Comparison of the total flow volume in ml of iNPH subgroups prior to STT with a good clinical response and after STT without clinical response with their standard deviations. Aqu – cerebral aqueduct, 4. V – 4. ventricle. C2 – second cervical vertebra. C4 – fourth cervical vertebra, 3. V – third ventricle.

As shown in Figure 5, no clear differences in flow volumes between iNPH patients with a clinical response before the STT and those without a clinical response after the STT. However, average flow volumes appeared to be higher in the iNPH group than in controls for intracranial regions, while the opposite was observed in extracranial regions. The differences between the post-STT to pre-STT mean flow volumes (*Δ*, in ml) were as follows: cerebral aqueduct: 1.47 mL (*p* = 0.657), fourth ventricle: 0.99 mL (*p* = 0.778), C2 level: 0.48 mL (*p* = 0.903), C4 level: 0.14 mL (*p* = 0.969) and third ventricle: 3.23 mL (*p* = 0.495). These consistent positive values may indicate a subtle overall increase in CSF-flow following the STT in clinically responsive patients.

### Correlations between CSF-flow volumes, clinical- and imaging parameters

Clinical parameters were correlated with CSF-flow volume measurements. A positive association was observed between flow volumes in intracranial ROIs and the Evans Index, as shown in Table 2. Additionally, a notable correlation between MoCA scores and TMT-A performance was found specifically at the level of the fourth cervical vertebra (Table 3). We found a negative correlation between the change in TUG test and change in CSF flow volume for the third ventricle (meaning an improvement in TUG test was associated with an increase in CSF flow volume). There was no significant correlations between the change in TUG and 30MWT and change in CSF flow volume in all other ROIs (see Supplementary Figures 1, 2). However, a consistent trend toward negative associations was observed between MoCA score and TMT-B performance across intracranial ROIs like the cerebral aqueduct, the 3^rd^ as well as the 4^th^ ventricle (Table 3).

**Table 2:** Correlation of flow volumes and radiological parameters.

Region of interest	Age (years)		Callosal angle		Evans index		Fazekas scale	
	*r*	*p*-value	*r*	*p*-value	*r*	*p*-value	*r*	*p*-value
Cerebral aqueduct (ml)	−0.386	0.093	−0.358	0.191	0.639	**0.006**	0.205	0.464
4^th^ ventricle (ml)	−0.366	0.124	−0.314	0.275	0.649	**0.007**	0.226	0.433
C2 (ml)	−0.247	0.295	−0.044	0.876	0.214	0.410	−0.212	0.295
C4 (ml)	−0.42	0.083	−0.039	0.898	0.036	0.898	−0.147	0.488
3^rd^ ventricle (ml)	−0.068	0.781	−0.344	0.228	0.586	**0.017**	0.283	0.325

This table shows a significant, positive correlation of intracranial flow volume and the Evans-index. Negative correlation of callosal angle and flow volume, but statistical significance not reached. Values in bold indicate statistical significance at *p* < 0.05.

**Table 3:** Correlation of flow volumes and cognitive tests.

Region of interest	MoCA (P)		TMT A (s)		TMT B (s)	
	*r*	*p*-value	*r*	*p*-value	*r*	*p*-value
Cerebral aqueduct (ml)	−0.111	0.651	−0.230	0.392	−0.341	0.214
4^th^ ventricle (ml)	−0.310	0.211	−0.158	0.560	−0.271	0.328
C2 (ml)	0.405	0.085	−0.246	0.358	0.025	0.929
C4 (ml)	0.623	**0.009**	−0.555	**0.039**	−0.368	0.217
3^rd^ ventricle (ml)	−0.412	0.089	0.043	0.873	−0.216	0.439

This table shows a significant, positive correlation of intracranial flow volume and the Evans-index. Negative correlation of callosal angle and flow volume, but statistical significance not reached. Values in bold indicate statistical significance at *p* < 0.05.

## Discussion

This exploratory study aimed to assess the diagnostic and predictive value of CSF-flow measurements using RT-PC MRI in patients with iNPH. By measuring flow volumes at specific intracranial and extracranial locations during a 40-s interval aligned with a breathing protocol, we compared data from iNPH patients to HC. Additionally, we evaluated changes pre- and post-STT and examined correlations with clinical assessments of cognition and motor function.

Our findings revealed that, on average, patients with iNPH exhibited higher intracranial total CSF-flow volumes compared to HC. However, these increased volumes did not correlate with clinical improvement following STT. Notably, a positive correlation was observed between flow volumes and a higher Evans index, suggesting that increased CSF-flow may be associated with disease progression rather than therapeutic response. Additionally, the CSF-flow volumes in the third ventricle are significantly higher and numerically elevated in other internal CSF spaces in iNPH patients, whereas flow volumes in the external CSF spaces do not differ from HC. Physiologically, CSF flow may normalize rapidly after lumbar puncture, and individual differences in ventricular compliance or circulation patterns may obscure transient effects. Elevated third ventricle flow likely reflects disease severity or ventricular enlargement rather than directly predicting responsiveness to CSF drainage or shunt placement.

Previous studies have explored the relationship between CSF-flow dynamics and clinical outcomes in iNPH, yet findings remain partially contradictory (Krauss et al., 1997; Hayhow et al., 2014). On the one hand, several studies reported a positive correlation between high CSF cardiac-dependent stroke volume, particularly at the level of the aqueduct, and favorable clinical outcomes following shunt implementation (Shanks et al., 2019). For instance, Scollato et al. (2008) demonstrated that all patients with aqueduct CSF stroke volumes greater than 42 μL responded favorably to shunting, indicating potential predictive value. The CSF stroke volume is commonly defined as the amount of CSF displaced during one complete cardiac cycle-comprising systole and diastole (Tawfik et al., 2017). Supporting these findings, different studies have shown that patients with increased preoperative stroke volumes tended to experience a decrease in flow following shunt placement, correlating with clinical improvement (Shanks et al., 2019; Yin et al., 2017; Qvarlander et al., 2017). Conversely, other research has reported conflicting results. A prospective study by Ringstad et al. (2015) found that while aqueduct CSF stroke volume was indeed elevated in iNPH patients and decreases after shunt surgery, there was no significant correlation between preoperative CSF-flow parameters and surgical outcomes, thus challenging its predictive reliability. Similarly, Chen et al. (2015) concluded that preoperative phase-contrast MRI CSF-flow parameters did not significantly differ between patients who improved after shunt surgery and those who did not, questioning their utility as predictive markers. Beyond cardiac influence, additional studies have underscored the importance of respiratory-driven CSF motion as a contributing factor to CSF-flow dynamics (Dreha-Kulaczewski et al., 2018; Dreha-Kulaczewski et al., 2015; Aktas et al., 2019; Yatsushiro et al., 2022).

In our exploratory study, the lack of a significant correlation between increased intracranial flow volumes and clinical improvement post-STT underscores the complexity of using CSF-flow measurements as predictive tools. The observed positive correlation with the Evans index aligns with the notion that increased CSF-flow may reflect disease severity rather than responsiveness to intervention. Additionally, the absence of significant findings in extracranial regions suggests that these areas may not be directly involved in the pathophysiological processes of iNPH. Moreover, the variability in individual patient anatomy, compliance with breathing protocols during imaging and the dynamic of CSF-flow influenced by cardiac and respiratory cycles could contribute to inconsistent results. Gait and cognitive performance were reassessed 24 h after CSF removal, which is why delayed responses occurring after this time frame may not have been captured and potential time-dependent fluctuations in CSF dynamics beyond this period cannot be excluded (Sakka et al., 2011). The timing and amount of CSF removed during the STT were not standardized, which may have influenced the variability in patient response. Age and sex distributions differed between iNPH and HC groups, which may have influences CSF flow parameters and group comparisons were therefore only possible to a limited extent. Although third ventricle CSF flow differed between iNPH patients and controls, no changes were observed pre- versus post-STT, limiting assessment of its diagnostic or predictive value. The absence of statistically significant change after STT should therefore be interpreted with caution, as the small sample size may have limited statistical power and a type II error cannot be excluded. Although patients were cognitively impaired (median MoCA 22 points), only those able to successfully complete the pre-scan respiratory training were included. Compliance with the respiratory protocol during MRI was assessed visually and via SpO₂ monitoring; however, full objective verification was not possible. ROI placement was performed consistently, with consultation of a second investigator in ambiguous cases. Therefore, the feasibility and reproducibility of this approach in broader patient populations remain to be validated. Aswell as the CSF volume removed during the STT in our study was at the lower end of the range reported in the literature (Singer et al., 2012), which may have contributed to a suboptimal STT response. Furthermore, the sample size of our study was modest (n = 15, controls = 5), and it was not designed as a confirmatory, adequately powered trial. *A priori* power calculations indicate that only very large effects (Cohen’s d ≈ 2.0) could be detected with 80% power in our sample; thus, our findings should be interpreted as preliminary and hypothesis-generating. Future research should include larger, adequately powered studies to confirm and expand upon these preliminary observations. However, strengths of this study include the careful selection of patients and the implementation of a novel imaging methodology.

In conclusion, while RT-PC MRI offers a non-invasive means to assess CSF dynamics, its current diagnostic and predictive value in iNPH remains uncertain. The significant inter-individual variability observed in flow volumes and the lack of correlation with clinical outcomes highlight the need for further research. Future studies with larger cohorts and standardized imaging protocols are essential to elucidate the role of CSF-flow measurements in the management of iNPH.

## Data Availability

The data analyzed in this study is subject to the following licenses/restrictions: dataset can be shared on request. Requests to access these datasets should be directed to ilko.maier@med.uni-goettingen.de.
