# Supplementary material for: CSF-flow prior and after spinal tap test in patients with idiopathic normal pressure hydrocephalus—an exploratory study using real-time phase-contrast MRI
Source: Front Neuroimaging. 2025 Dec 12;4:1665687. doi: 10.3389/fnimg.2025.1665687 (PMC12740890; doi:10.3389/fnimg.2025.1665687)

<u>Supplementary Figures:</u>

Supplementary Figure 1: Scatter plot of ΔCSF-flow and Δ of Timed-up-and-go Test of iNPH patients in four different positions: Cerebral aqueduct, 4th ventricle, C2, C4 and the 3rd ventricle.

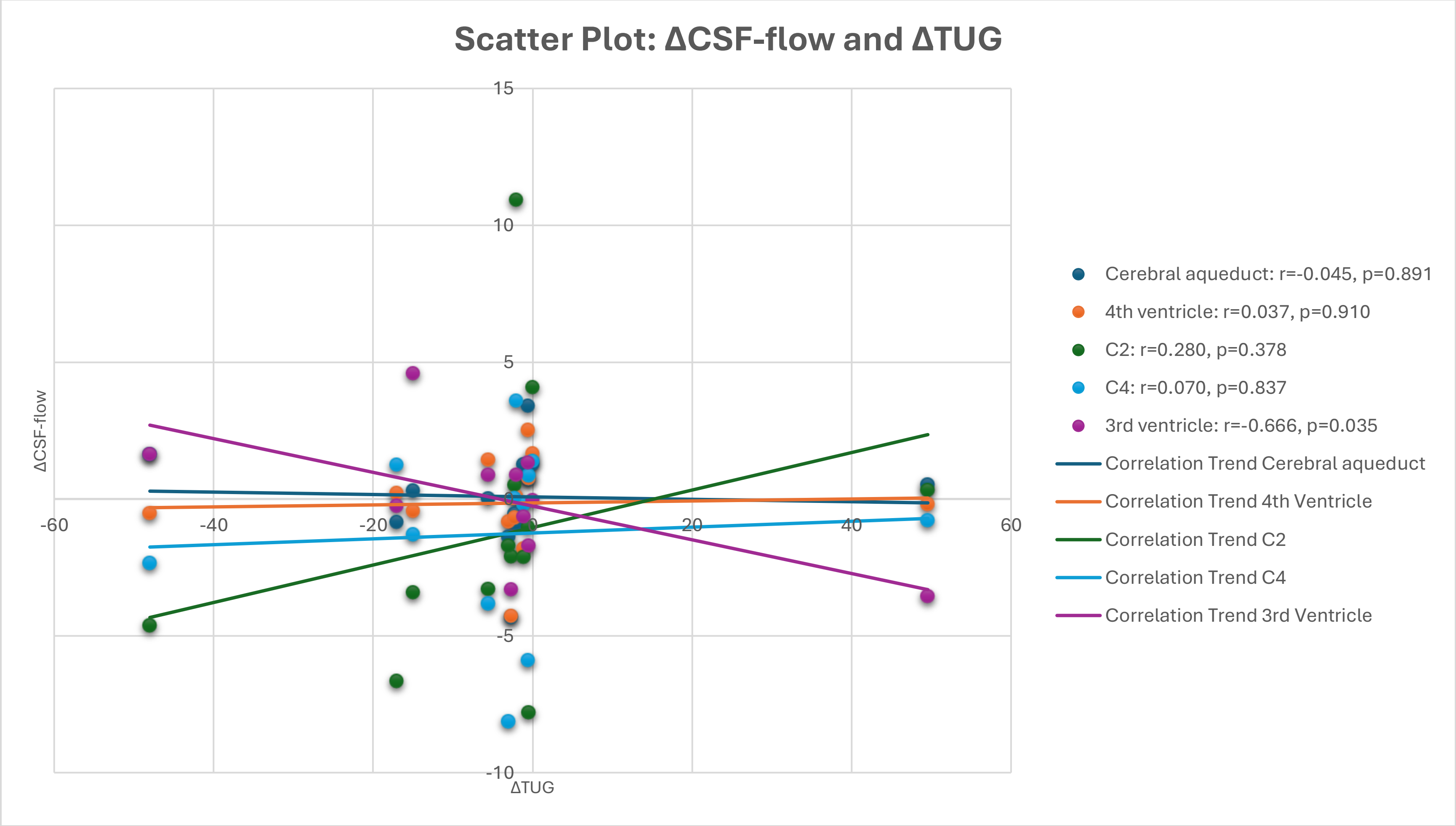

CSF - cerebrospinal fluid, TUG - Timed-up-and-go Test*, C2 – second cervical vertebra, C4 – fourth cervical vertebra*

Supplementary figure 2: Scatter plot of ΔCSF-flow and Δ of 30-Meter-Walk-Test of iNPH patients in four different positions: Cerebral aqueduct, 4th ventricle, C2, C4 and the 3rd ventricle.

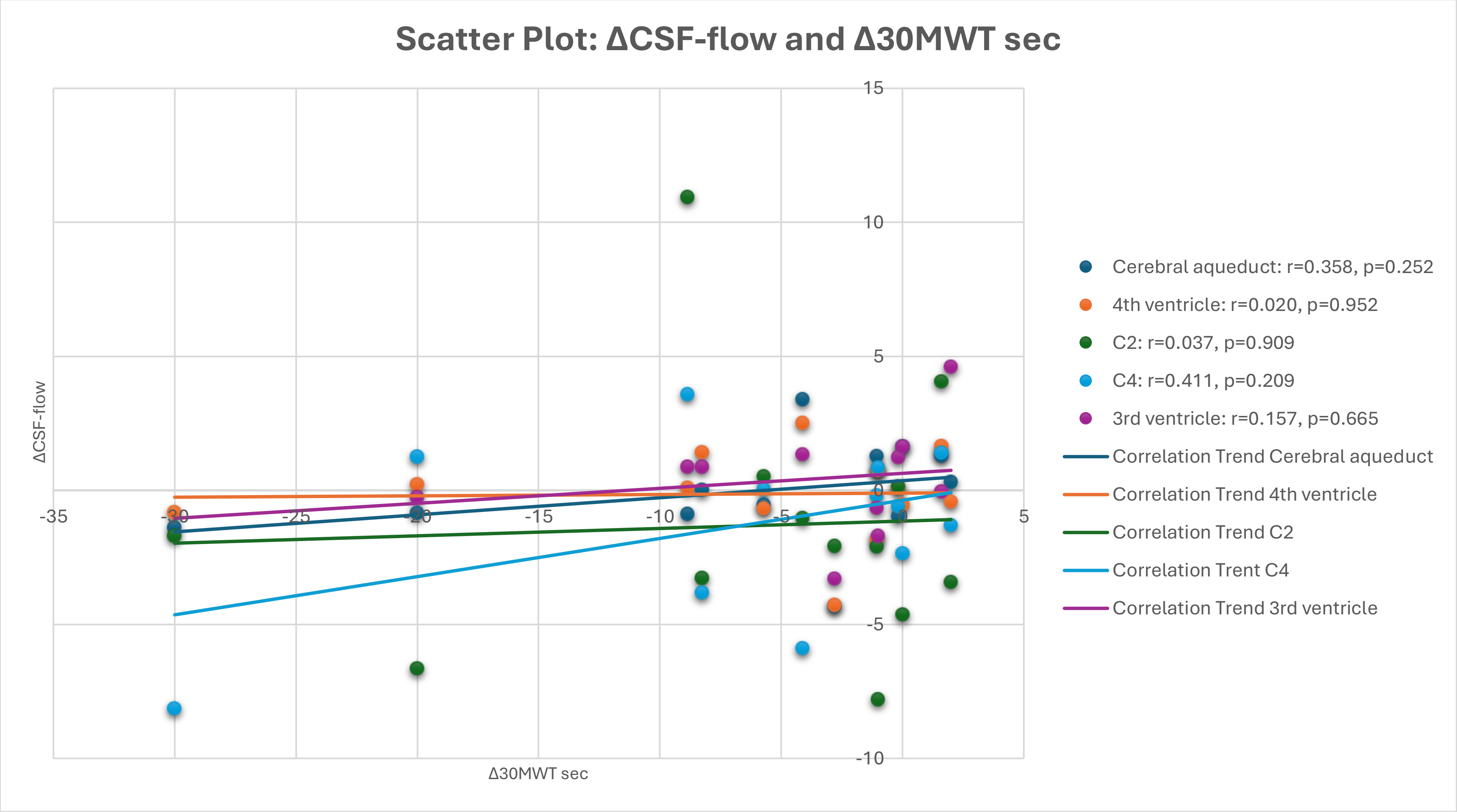

CSF - cerebrospinal fluid, 30MWT - 30-Meter-Walk-Test, C2 – second cervical vertebra C4 – fourth cervical vertebra

Supplementary figure 3:
ROC curve for baseline 3rd ventricle volume (ml) in differentiating iNPH from control group patients.

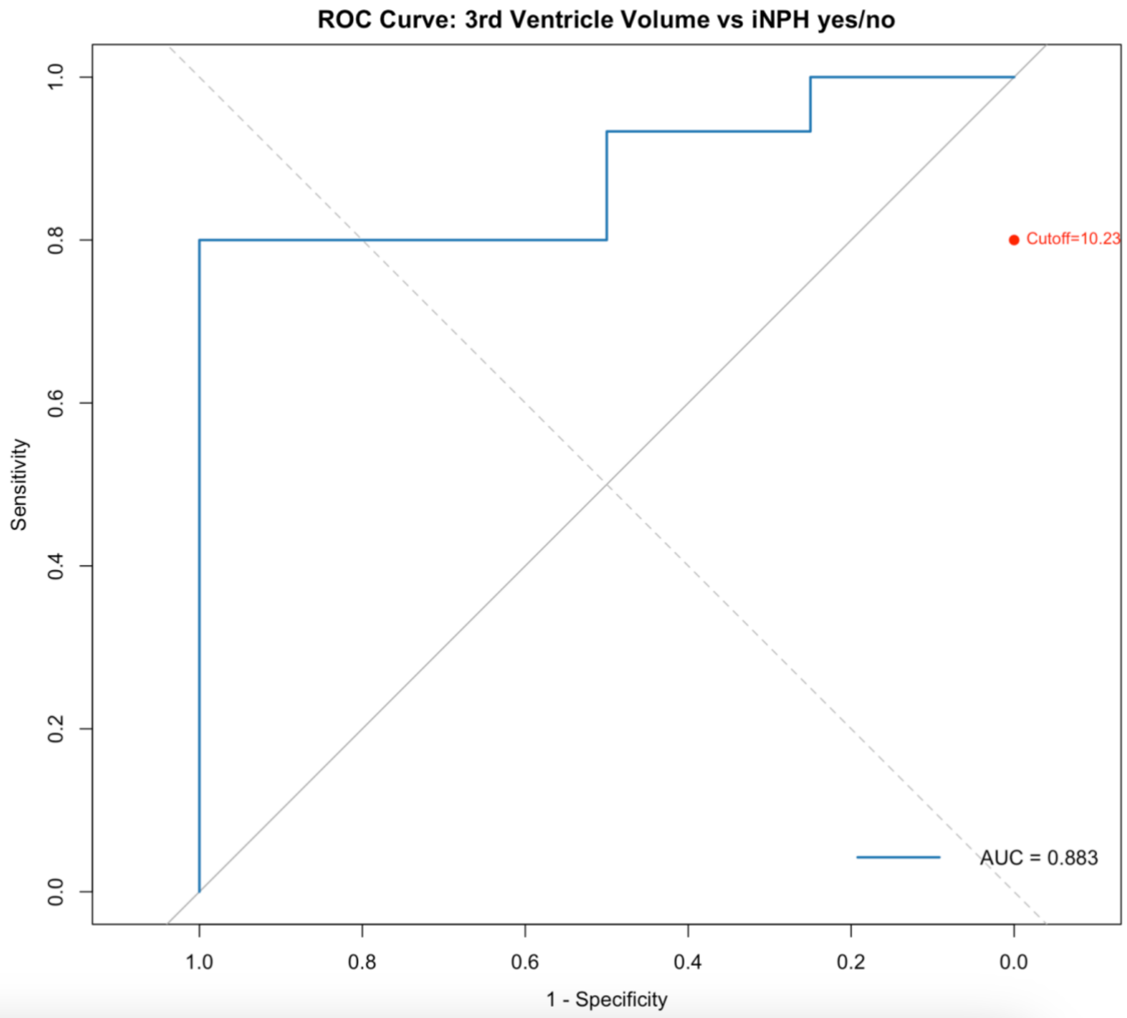

Supplement: Supplementary file 1 [file Data_Sheet_1.PDF]
